# Lung ultrasound for the diagnosis of cystic fibrosis pulmonary exacerbation

**DOI:** 10.1186/s12890-021-01728-8

**Published:** 2021-11-08

**Authors:** Maryam Hassanzad, Arda Kiani, Atefeh Abedini, Hoseinali Ghaffaripour, Habib Emami, Niloufar Alizadeh, Ghazal Zoghi, Saeed Hashemi, Ali Akbar Velayati

**Affiliations:** 1 Pediatric Respiratory Diseases Research Center (PRDRC), National Institute of Tuberculosis and Lung Diseases (NRITLD), Shahid Beheshti University of Medical Sciences, Tehran, Iran; 2 Chronic Respiratory Diseases Research Center, National Institute of Tuberculosis and Lung Diseases (NRITLD), Shahid Beheshti University of Medical Sciences, Tehran, Iran; 3 Tobacco Prevention and Control Research Center, National Institute of Tuberculosis and Lung Diseases (NRITLD), Shahid Beheshti University of Medical Sciences, Tehran, Iran; 4 Department of Biostatistics, National Institute of Tuberculosis and Lung Diseases (NRITLD), Shahid Beheshti University of Medical Sciences, Tehran, Iran; 5 Endocrinology and Metabolism Research Center, Hormozgan University of Medical Sciences, Bandar Abbas, Iran; 6 Mycobacteriology Research Center (MRC), National Institute of Tuberculosis and Lung Diseases (NRITLD), Shahid Beheshti University of Medical Sciences, Tehran, Iran

**Keywords:** Cystic fibrosis, Pulmonary exacerbation, Ultrasound

## Abstract

**Background:**

High-resolution computed tomography (HRCT) is the gold standard for the evaluation of cystic fibrosis (CF) lung disease; however, lung ultrasound (LUS) is being increasingly used for the assessment of lung in these patients due to its lower cost, availability, and lack of irradiation. We aimed to determine the diagnostic performance of LUS for the evaluation of CF pulmonary exacerbation.

**Methods:**

This cross-sectional study included patients with CF pulmonary exacerbation admitted to Masih Daneshvari Hospital, Tehran, Iran, from March 21, 2020 to March 20, 2021. Age, gender, and body mass index (BMI) of the patients were recorded. All patients underwent chest X-ray (CXR), HRCT, and LUS on admission. Pleural thickening, atelectasis, air bronchogram, B-line, and consolidation were noted in LUS and then compared with the corresponding findings in CXR and HRCT. Taking HRCT findings as reference, sensitivity, specificity, positive predictive value (PPV), negative predictive value (NPV), and diagnostic accuracy (DA) of LUS and CXR for the detection of each pulmonary abnormality were determined.

**Results:**

Of the 30 patients included in this study, with a mean age of 19.62 ± 5.53 years, 14 (46.7%) were male. Of the 15 patients aged 2–20 years, BMI was below the 5^th^ percentile in 10 (66.7%), within the 5–10 percentiles in 1 (6.7%), 10–25 percentiles in 3 (20%), and 25-50 percentiles in 1 (6.7%). The mean BMI for 15 patients > 20 years was 18.03 ± 2.53 kg/m^2^. LUS had better diagnostic performance compared to CXR for the detection of air bronchogram, consolidation, and pleural thickening (area under the receiver operating characteristic curve [AUROC]: 0.966 vs. 0.483, 0.900 vs. 0.575, and 0.656 vs. 0.531, respectively). Also, LUS was 100% and 96.7% specific for the diagnosis of pleural effusion and atelectasis, respectively.

**Conclusions:**

LUS appears to be superior to CXR and comparable with HRCT for the evaluation of CF pulmonary exacerbation, especially in terms of air bronchogram and consolidation detection. LUS can be used to lengthen the HRCT evaluation intervals in this regard or utilized along with HRCT for better evaluation of CF pulmonary exacerbation.

## Introduction

Cystic fibrosis (CF) is a progressive genetic disease caused by a single-gene mutation, resulting in chemical change in the cystic fibrosis transmembrane conductance regulator (CFTR), a protein that forms a chloride channel with a critical role in mucus transportation [1]. The prevalence of CF has been estimated at 1 in 100,000 in the Iranian population [2].

CF lung disease can present with frequent lung infections, recurrent wheezing, tachypnea, and persistent coughing. The onset of CF lung disease is highly variable; however, respiratory manifestations do not commonly develop until later infancy [3]. CF lung disease occurs as a result of recurring cycles of inflammation and infection, culminating in chronic damage to the lung parenchyma which progresses to respiratory failure and even death [4–6]. There is no consensus regarding the definition of CF pulmonary exacerbation; nonetheless, exacerbations are usually well recognized by the acute worsening of signs and symptoms, as well as deterioration of CF lung disease and transient decline in forced expiratory volume in 1 s (FEV_1_) [7].

Although various tools have been used to evaluate CF lung disease, high-resolution computed tomography (HRCT) remains the gold standard as it allows quantitative and qualitative evaluation of the lung [8]. Nevertheless, radiation exposure and the necessity of anesthesia in younger children, together with its high cost, limit the use of HRCT in CF patients [9]. Magnetic resonance imaging (MRI) is another modality that has recently been proposed for life-long imaging surveillance of CF patients, with the advantage of lacking ionizing radiation [10]. Ultrasound is currently the most commonly used imaging technique; therefore, lung ultrasound (LUS) can be an important tool in the evaluation of children with CF having multiple advantages, including availability, cost-effectiveness, non-invasiveness, safety, and bedside usability in critically ill patients [11, 12]. In the current study, we aimed to determine the diagnostic performance of LUS in the evaluation of CF pulmonary exacerbation.

## Methods

### Participants

This cross-sectional study included patients with CF admitted to Masih Daneshvari Hospital, Tehran, Iran, from March 21, 2020 to March 20, 2021. Inclusion criteria were signs and symptoms of CF pulmonary exacerbation, including fever, tachypnea, respiratory distress, worsened cough, increased sputum, decreased appetite, weight loss, decreased saturation, and the like [13]. Adult patients and pediatric patients whose parents/guardians did not consent to participate in the study were excluded.

### Study design

General features including age, gender, and body mass index (BMI) were recorded for each patient. All patients underwent chest X-ray (CXR) and HRCT (64-channel multidetector CT scanner, SOMATOM go.Up, Siemens Healthineers, Germany) on their first day of admission. Also, LUS was performed on admission by an expert pulmonologist for all patients, using Philips ultrasound device (Philips Healthcare Co., Taiwan). The pulmonologist was blinded to CXR and HRCT findings. For LUS, each hemithorax was divided into 3 parts, including anterior, lateral, and posterior areas, demarcated by the posterior axillary line, the anterior axillary line, and the parasternal line [14, 15]. The convex probe was placed perpendicular to the thorax and moved parallel to the ribs to evaluate each intercostal space. The patient’s position was supine for the evaluation of the anterior area, lateral decubitus for the lateral area, and prone for the posterior area. Pleural thickening, atelectasis, air bronchogram, B-line, and consolidation were noted in LUS and then compared with the corresponding findings in CXR and HRCT. Taking the findings of HRCT as reference, the diagnostic performance of LUS and CXR for each finding was determined.

### Data analysis

We used the Statistical Package for the Social Sciences (SPSS) software (version 26.0, IBM Corp., Armonk, NY, USA) for data analysis. Mean and standard deviation were used to describe quantitative variables. Frequencies and percentages were used to describe qualitative variables. The receiver operating characteristic (ROC) curves of LUS and CXR were drawn for each pulmonary finding based on CT-scan results. Accordingly, the area under the curve (AUC) value from the ROC curves were calculated. Also, taking the CT-scan results into account, sensitivity, specificity, positive predictive value (PPV), negative predictive value (NPV), and diagnostic accuracy (DA) of both CXR and LUS for the diagnosis of different pulmonary findings were calculated. *P* values ≤ 0.05 were regarded as statistically significant.

## Results

**Fig. 1:**
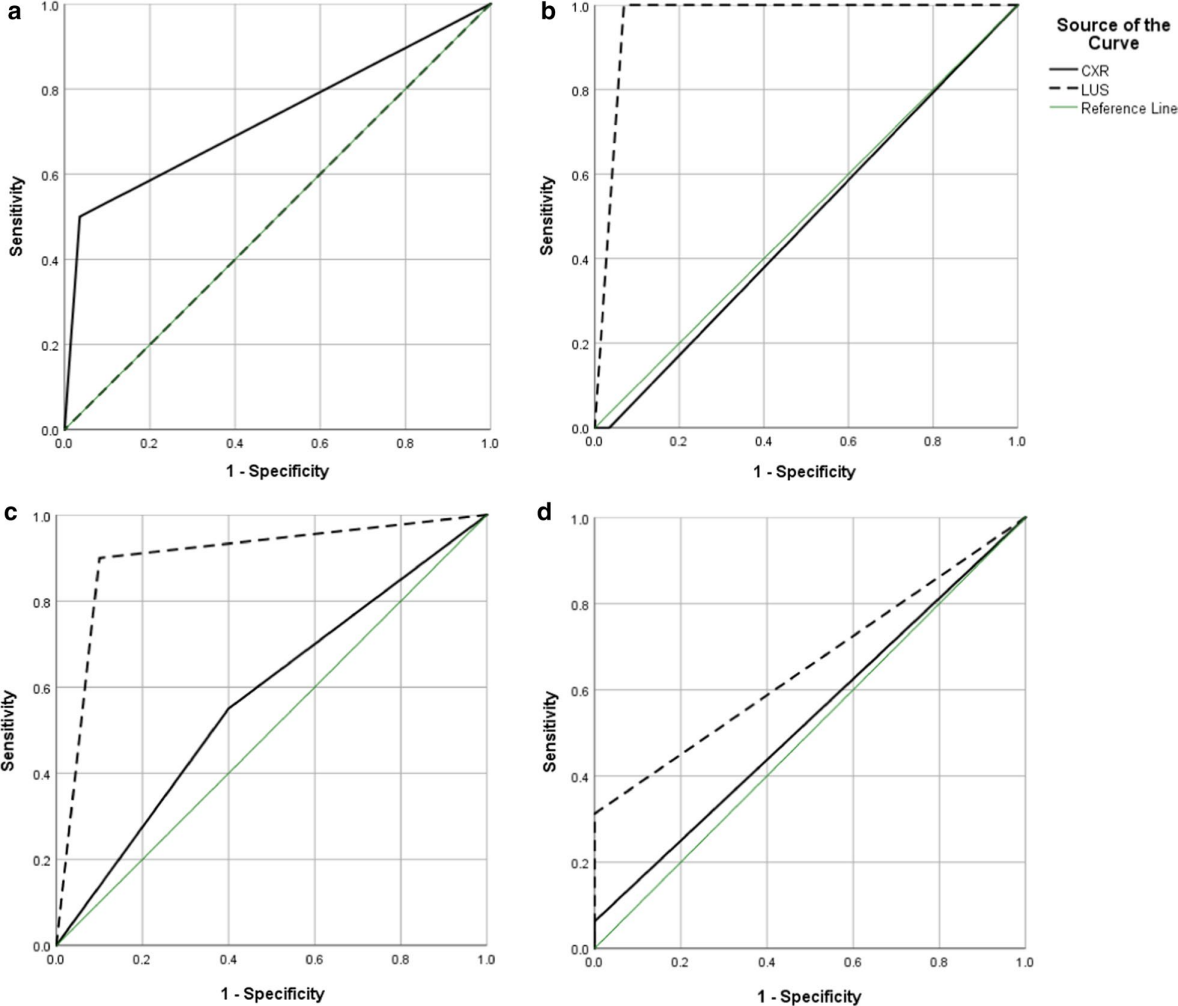
ROC curves of LUS and CXR for the detection of different pulmonary abnormalities: **a** atelectasis; **b** air bronchogram; **c** consolidation; and **d** pleural thickening

Of the 30 patients included in this study, with a mean age of 19.62 ± 5.53 (range: 6–29) years, 14 (46.7%) were male and 16 (53.3%) were female. Of the 15 patients aged 2–20 years, BMI was below the 5^th^ percentile in 10 (66.7%), within the 5–10 percentiles in 1 (6.7%), 10–25 percentiles in 3 (20%), and 25–50 percentiles in 1 (6.7%). The mean BMI for 15 patients > 20 years was 18.03 ± 2.53 kg/m^2^. Pleural effusion was detected in none of the patients using HRCT, while it was detected in 1 patient using LUS and CXR. Therefore, the sensitivity of LUS and CXR for the diagnosis of pleural effusion was incalculable and ROC curves could not be drawn. LUS had 100% specificity for the detection of atelectasis (Table 1). The best diagnostic performance belonged to LUS for the detection of consolidation with 94.7% sensitivity, 90% specificity, 94.7% PPV, 81.8% NPV, 90% DA, and an AUC of 0.900 (95% CI 0.766–1.000, *P* < 0.001). LUS was 100% sensitive for the detection of air bronchogram. In addition, both LUS and CXR had 100% specificity for the diagnosis of pleural thickening (Table 1). Figure 1 demonstrates the ROC curves of LUS and CXR for the detection of atelectasis, air bronchogram, consolidation, and pleural thickening.

**Table 1:** Comparison of imaging findings in patients with CF pulmonary exacerbation by different modalities

Findings	HRCT			Diagnostic performance						
	Positive (N)	Negative (N)	Total (N)	Sensitivity (%)	Specificity (%)	PPV (%)	NPV (%)	DA (%)	AUC (95% CI)	*P* value
Pleural effusion										
LUS (N)				Incalculable	96.7	0	100.0	96.7	–	–
Positive	0	1	1							
Negative	0	29	29							
Total	0	30	30							
CXR (N)				Incalculable	96.7	0	100.0	96.7	–	–
Positive	0	1	1							
Negative	0	29	29							
Total	0	30	30							
Atelectasis										
LUS (N)				0	100.0	93.3	Incalculable	93.3	0.500 (0.079–0.921)	1.000
Positive	0	0	0							
Negative	2	28	30							
Total	2	28	30							
CXR (N)				50	96.4	50	96.4	93.3	0.732 (0.281–1.000)	0.280
Positive	1	1	2							
Negative	1	27	28							
Total	2	28	30							
Air bronchogram										
LUS (N)				100.0	93.1	33.3	100.0	93.3	0.966 (0.889–1.000)	0.119
Positive	1	2	3							
Negative	0	27	27							
Total	1	29	30							
CXR (N)				0	96.6	0	96.6	93.3	0.483 (0.000–1.000)	0.954
Positive	0	1	1							
Negative	1	28	29							
Total	1	29	30							
Consolidation										
LUS (N)				94.7	90.0	94.7	81.8	90.0	0.900 (0.766–1.000)	< 0.001
Positive	18	1	19							
Negative	2	9	11							
Total	20	10	30							
CXR (N)				73.3	60.0	73.3	40.0	56.7	0.575 (0.355–0.795)	0.509
Positive	11	4	15							
Negative	9	6	15							
Total	20	10	30							
Pleural thickening										
LUS (N)				31.3	100.0	100.0	56.0	63.3	0.656 (0.459–0.853)	0.146
Positive	5	0	5							
Negative	11	14	25							
Total	16	14	30							
CXR (N)				6.3	100.0	100.0	48.3	50.0	0.531 (0.322–0.741)	0.771
Positive	1	0	1							
Negative	15	14	29							
Total	16	14	30							

N, Number; LUS, lung ultrasound; CXR, chest X-ray; HRCT, high-resolution computed tomography; PPV, positive predictive value; NPV, negative predictive value; DA, diagnostic accuracy; AUC, area under the curve; CI, confidence interval

The area under the ROC curve of LUS for the detection of B-lines as the corresponding finding of subpleural opacity/septal thickening in HRCT was 0.611 (Table 2). Also, the area under the ROC curve of LUS for the detection of B-lines, taking subpleural opacity/septal thickening or consolidations as their equivalent in HRCT, was 0.397 (Table 3).

**Table 2:** Comparison of LUS and HRCT regarding subpleural opacity/septal thickening

Findings	Subpleural opacity/septal thickening in HRCT			Diagnostic performance						
	Positive (N)	Negative (N)	Total (N)	Sensitivity (%)	Specificity (%)	PPV (%)	NPV (%)	DA (%)	AUC (95% CI)	*P* value
B-line										
LUS (N)				88.9	33.3	66.7	66.7	66.7	0.611 (0.397–0.825)	0.310
Positive	16	8	24							
Negative	2	4	6							
Total	18	12	30							

N, Number; LUS, lung ultrasound; HRCT, high-resolution computed tomography; PPV, positive predictive value; NPV, negative predictive value; DA, diagnostic accuracy; AUC, area under the curve; CI, confidence interval

**Table 3:** Comparison of LUS and HRCT regarding subpleural opacity/septal thickening/consolidation

Findings	Subpleural opacity/septal thickening/consolidation in HRCT			Diagnostic performance						
	Positive (N)	Negative (N)	Total (N)	Sensitivity (%)	Specificity (%)	PPV (%)	NPV (%)	DA (%)	AUC (95% CI)	*P* value
B-line										
LUS (N)				79.3	0.0	95.8	0.0	76.7	0.397 (0.000–0.872)	0.729
Positive	23	1	24							
Negative	6	0	6							
Total	29	1	30							

N, Number; LUS, lung ultrasound; HRCT, high-resolution computed tomography; PPV, positive predictive value; NPV, negative predictive value; DA, diagnostic accuracy; AUC, area under the curve; CI, confidence interval

## Discussion

We found that LUS and CXR were comparable regarding the evaluation of pleural effusion in patients with CF pulmonary exacerbation. On the other hand, LUS was superior to CXR for the detection of air bronchogram, consolidation, and pleural thickening. CXR was only superior to LUS when the diagnosis of atelectasis was concerned. Furthermore, LUS did not yield an acceptable diagnostic performance for the detection of subpleural opacity/septal thickening.

Progressive lung disease limits the survival of CF patients. Having a proper attitude towards the severity of CF lung disease and monitoring it over time is important in directing clinical care and predicting disease outcomes. Imaging provides information about the regional distribution of CF lung disease; therefore, pulmonary imaging studies are recommended for the follow-up of CF patients. CXR, HRCT, and MRI are currently the available imaging techniques [16, 17]. CXR is routinely used in CF clinics and is usually performed annually. Although CXR is valuable for the long-term monitoring of CF patients, it has limited sensitivity when it comes to early pulmonary abnormalities, such as air trapping and primary bronchiectasis [17]. HRCT can demonstrate the initial abnormalities, as well as the progression of structural changes, while delineating the highest morphologic details [18]. HRCT is the gold standard for the diagnosis of pulmonary lesions; nevertheless, its routine use in CF patients, the majority of whom are young and sensitive to ionizing radiation, and its life-long accumulation, is debatable [18]; yet, low-dose chest CT has been proposed as a solution to reduce radiation exposure in a recent study. It has been reported to yield satisfactory image quality with diagnostic capabilities equivalent to standard CT [19]. On the other hand, CXR also exposes the patients to ionizing radiation. In recent years, ultrasound has been used for the evaluation and monitoring of intensive care unit patients. LUS can be used at bed side and it is also rapid, inexpensive, and non-ionizing [20]. Moreover, LUS is easier to use in infants and children [21]. This is of significance since the percentage of pediatric CF patients is higher than adults in the Iranian population [22], unlike many other countries, such as the European countries, where adults make up the majority of CF patients [23].

Consistent with our findings, Peixoto et al. assessed the applicability of ultrasound for the evaluation of CF lung disease and showed that LUS results were comparable with HRCT [24]. Also, Ciuca et al. suggested LUS as a monitoring tool for CF lung disease and pulmonary exacerbation [25]. In another study by Ciuca et al., LUS showed typical signs of consolidation in 8.5% of CF patients [26]. LUS was able to detect consolidation in 19 (63.3%) patients of our study. The discrepancy between their study and ours may be explained by different degrees of exacerbation severity and the etiology behind this condition in the two studies. Ciuca et al. also showed acceptable correlations between CT findings and LUS regarding the detection of consolidation [26]. Our results were in line with their findings. We also showed a good diagnostic performance for the detection of consolidation by LUS (AUC of 0.900).

LUS has also been used for the diagnosis of pneumonia in previous studies. For instance, Berce et al. showed the applicability of LUS for distinguishing bacterial community-acquired pneumonia (CAP) from CAP of other etiologies [27]. Musolino et al. compared LUS findings in children with complicated CAP to those with uncomplicated CAP. In their study, subpleural parenchymal lesions, pleural effusion, and bronchogram were evaluated with LUS, which shows the efficacy of LUS for the detection of these abnormalities [28]. Moreover, Yilmaz et al. demonstrated the comparability of LUS with CXR for the diagnosis of CAP in children [29]. However, in the current study, LUS was superior to CXR for the detection of the majority of CF pulmonary exacerbation findings.

In another study, Dankoff et al., evaluated LUS findings in children with asthma who had been admitted to the emergency department due to respiratory distress. In this study, B-line, consolidation, and pleural line abnormalities were found in 38%, 30%, and 12% of asthma patients, respectively [30]. Accordingly, LUS appears to be an appropriate tool for the evaluation of lung in asthma exacerbation as well. Ho et al. also found LUS to be a sensitive tool for the diagnosis and follow-up of pneumonia in children [31]).

Our study was not without limitations. One limitation was our small sample size which did not allow us to draw ROC curves for either LUS or CXR for the detection of pleural effusion. With the low prevalence of CF, a multi-center study would have solved the problem, while adding another limitation which would be the potential interobserver variability for LUS evaluations at different centers. Another limitation is that we were not able to perform spirometry to determine the severity of the disease based on FEV_1_ due to the coronavirus disease 2019 pandemic and the possibility of disease transmission. Furthermore, although dornase alfa was not available for the patients and none of them received this medication, all patients underwent chest physiotherapy at home, which may have interfered with imaging findings.

## Conclusions

In this study LUS was superior to CXR for the detection of air bronchogram, consolidation, and pleural thickening in patients with CF pulmonary exacerbation. However, the same was not true for pleural effusion and atelectasis. LUS can be used together with HRCT for better evaluation of these patients. Based on our results, there is the possibility of replacing CXR with LUS in the clinical urgency of respiratory worsening in CF patients. Also, LUS can be used to follow up the response to therapy in pulmonary exacerbations. Nevertheless, future studies with a larger sample size are required to confirm our findings.

## Data Availability

The datasets used and/or analyzed during the current study are available from the corresponding author on reasonable request.

## Abbreviations

AUC: Area under the curve
AUROC: Area under the receiver operating characteristic curve
BMI: Body mass index
CAP: Community-acquired pneumonia
CF: Cystic fibrosis
CFTR: Cystic fibrosis transmembrane conductance regulator
CT: Computed tomography
CXR: Chest X-ray
DA: Diagnostic accuracy
FEV_1_: Forced expiratory volume in 1 s
HRCT: High-resolution computed tomography
LUS: Lung ultrasound
MRI: Magnetic resonance imaging
NPV: Negative predictive value
PPV: Positive predictive value
ROC: Receiver operating characteristic
